# An extended 36-week oral esomeprazole improved long-term recurrent peptic ulcer bleeding in patients at high risk of rebleeding

**DOI:** 10.1186/s12876-022-02534-0

**Published:** 2022-10-21

**Authors:** Hsueh-Chien Chiang, Er-Hsiang Yang, Huang-Ming Hu, Wei-Ying Chen, Wei-Lun Chang, Chung-Tai Wu, Deng-Chyang Wu, Bor-Shyang Sheu, Hsiu-Chi Cheng

**Affiliations:** 1 Department of Internal Medicine, National Cheng Kung University Hospital, College of Medicine, National Cheng Kung University, 138 Sheng Li Rd, Tainan, 704302 Taiwan; 2 Division of Gastroenterology, Department of Internal Medicine, Kaohsiung Medical University Hospital, Kaohsiung Medical University, 100, Ziyou 1st Rd, Kaohsiung, 807377 Taiwan; 3 Department of Internal Medicine, Kaohsiung Municipal Ta-Tung Hospital, 68, Zhonghua 3rd Rd, Kaohsiung, 801735 Taiwan; 4 Department of Public Health, College of Medicine, National Cheng Kung University, 138 Sheng Li Rd, Tainan, 704302 Taiwan; 5 Institute of Clinical Medicine, College of Medicine, National Cheng Kung University, 138 Sheng Li Road, North Dist, Tainan, 704302 Taiwan; 6 School of Medicine, College of Medicine, Kaohsiung Medical University Hospital, Kaohsiung Medical University, 100, Ziyou 1st Rd, Kaohsiung, 807377 Taiwan; 7 Institute of Molecular Medicine, College of Medicine, National Cheng Kung University, 138 Sheng Li Road, North Dist, Tainan, 704302 Taiwan; 8 Department of Internal Medicine, Tainan Hospital, Ministry of Health and Welfare, 125 Jhongshan Road, West Central Dist, Tainan, 700007 Taiwan

**Keywords:** Peptic ulcer hemorrhage, Stomach ulcer, Duodenal ulcer, Proton pump inhibitors, Risk scores

## Abstract

**Background:**

Patients with Rockall scores ≥6 have an increased risk of long-term peptic ulcer rebleeding. This study was aimed toward investigating whether an extended course of oral esomeprazole up to 1 year decreased ulcer rebleeding in such patients.

**Methods:**

We prospectively enrolled 120 patients with peptic ulcer bleeding and Rockall scores ≥6. After an initial 16-week oral proton pump inhibitor (PPI) treatment, patients were randomized to receive a 36-week course of oral twice-daily esomeprazole 20 mg (Group D, *n* = 60) or once-daily (Group S, *n* = 60). Thereafter, they were divided into the PPI-on-demand (*n* = 32) and PPI-discontinued (*n* = 77) subgroups. Our previous cohort with Rockall scores ≥6 served as the controls (Group C, *n* = 135); they received only an initial 8- to 16-week oral PPI. The primary and secondary outcomes were peptic ulcer rebleeding during the first year and the second year-and-thereafter, respectively.

**Results:**

For the primary outcome, groups D and S comprised a higher proportion of rebleeding-free than Group C (*P* = 0.008 and 0.03, log-rank test). The competing-risks regression analysis confirmed that extended PPI use and American Society of Anesthesiologists classification were independent factors contributing to the primary outcome. For the secondary outcome, PPI-on-demand had a borderline higher proportion of rebleeding-free than Group C (*P* = 0.07, log-rank test); however, only the Rockall score was the independent factor.

**Conclusions:**

An extended 36-week course of oral esomeprazole 20 mg, twice- or once-daily for patients with Rockall scores ≥6 reduced ulcer rebleeding during the first year, but the effect needed to be further validated when PPIs were shifted to on-demand or discontinued thereafter (NCT02456012, 28/05/2015).

**Supplementary Information:**

The online version contains supplementary material available at 10.1186/s12876-022-02534-0.

## Background

Peptic ulcer bleeding is important in clinical practice because it is a common issue and is correlated with mortality [1–4]. Recurrent bleeding events not only develop within 30 days after the first bleeding episode but also over subsequent years. About one-third of patients with peptic ulcer bleeding have recurrent bleeding episodes within the first 1–2 years and have increased episodes over the subsequent 10 years [5]. The Rockall score has been widely used in predicting recurrent ulcer bleeding [3, 4]. The Rockall score contains five parameters with total scores from 0 to 11, including age, shock, comorbidity, endoscopic diagnosis, and endoscopic evidence of bleeding (Supplementary Table 1) [6, 7]. Previous studies showed that patients with Rockall scores ≥6 had an increased risk of short-term and long-term recurrent ulcer bleeding [6–9].

The use of proton pump inhibitors (PPIs) is the current standard treatment for peptic ulcer disease with bleeding because PPIs heal peptic ulcers well and decrease the risk of recurrent bleeding [10–12]. However, it is still uncertain as to whether to prescribe a prolonged course of PPIs for patients who are at risk of long-term recurrent peptic ulcer bleeding except those with antiplatelet agent use [3, 11, 12]. In addition, the suppression of gastric acid secretion may be incomplete when taking oral PPIs only once-daily [13, 14]. Therefore, based on the adage for medication use (the lowest effective dose and the shortest possible time) [15], this study was aimed toward validating the effectiveness of oral esomeprazole at two different dosage increments as compared to controls in an effort to reduce the risk of peptic ulcer rebleeding among high-risk patients whose Rockall scores at baseline were ≥ 6. In addition, an attempt was made to compare the effectiveness when oral esomeprazole was used during the first year and discontinued thereafter.

## Methods

### Study design and setting

**Fig. 1:**
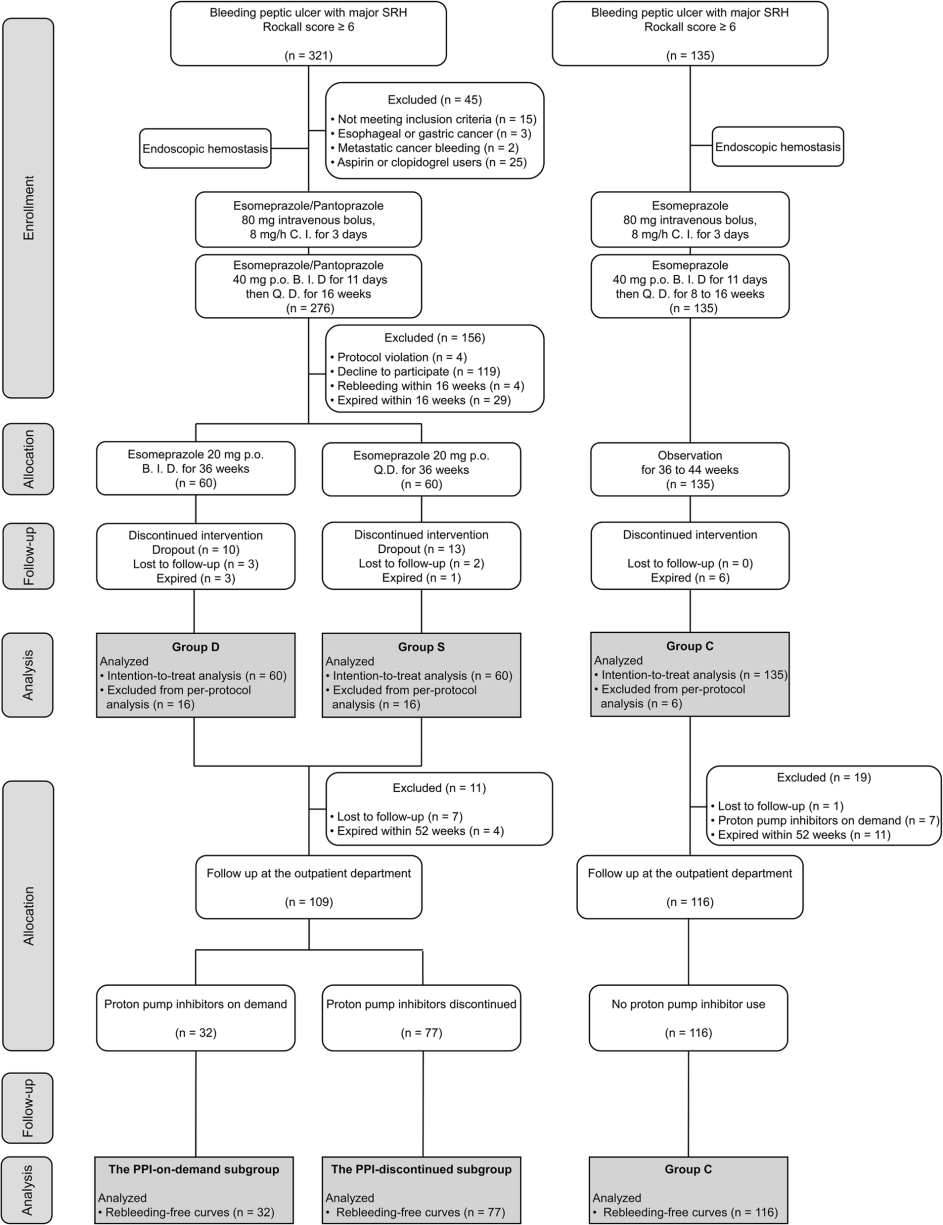
Trial profile

This prospective study was conducted in the inpatient wards of two tertiary healthcare centers, National Cheng Kung University Hospital in Tainan City and Kaohsiung Medical University Hospital in Kaohsiung City, Taiwan from June 2015 to December 2020. The study design was approved by the National Cheng Kung University Hospital Institutional Review Board (approval number: A-BR-104-007 and trial registration identifier: NCT02456012, ClincalTrials.gov, 28/05/2015). After they provided written informed consent, the participants were enrolled. A schematic flow chart of the study protocol is summarized in Fig. 1.

### The characteristics of participants

Patients 20 or more years of age who were diagnosed with bleeding peptic ulcers with major stigmata of recent hemorrhage (SRH) were considered eligible. The major SRH include Forrest Ia, Ib, IIa, and IIb [16]. At the index endoscopy, all patients received one or a combination of endoscopic therapies to stop bleeding. The endoscopic therapy methods were listed in our previous study in detail [9].

In this study, we used *Rockall scores ≥ 6* to define patients who were at high risk of rebleeding [6–9]. The parameters of the Rockall score are listed in Supplementary Table 1 and the definitions for the co-morbidities in the score were listed in previous studies in more detail [6, 9]. After gastroscopy to confirm enrollment eligibility, only patients with Rockall scores ≥6 were enrolled. The exclusion criteria were patients with bleeding from marginal ulcers, tumors, Dieulafoy lesions, or mechanical factor-related ulcers (i.e., gastrostomy tube induction), or with hypersensitivity to esomeprazole or pantoprazole. Patients who had used or planned to use long-term antiplatelet agents (i.e., aspirin, clopidogrel, ticagrelor, or prasugrel) or used non-steroidal anti-inflammatory drugs after the first bleeding episode were also excluded because the protective effect of PPIs for such patients has been established [11, 12].

Our study group had a cohort in which patients with bleeding peptic ulcer diseases and Rockall scores ≥6 from Aug 2011 to Jan 2015 were enrolled [8, 9]. The patients in this cohort received an 8- to 16-week treatment with oral PPIs after the first peptic ulcer bleeding episode. From this cohort, patients who were age- and sex-matched for the experimental groups (Group D and S) were chosen as the controls, which were labeled as Group C.

American Society of Anesthesiologists (ASA) Physical Status classification (Supplementary Table 2) is a simple tool to summarize the preoperative health status of surgical patients; moreover, it has become a ubiquitous component of clinical studies to define and compare demographic characteristics of subjects between groups [17].

### Randomization and masking

Each patient received intravenous PPI therapy with an 80 mg loading dose and then an 8 mg/h continuous infusion of either esomeprazole (Nexium®, AstraZeneca AB, Södertälje, Sweden) or pantoprazole (Pantoloc®, Takeda, Singen, Germany) for 3 days [1]. Thereafter, the patients received oral esomeprazole or pantoprazole 40 mg twice a day for the following 11 days and once a day for the remaining days within the first 16 weeks [9]. After the first 16-week PPI therapy, patients were randomized into Group D or Group S following a simple randomization procedure with a 1:1 allocation ratio based on drawing an envelope from a box of sealed opaque envelopes, each of which contained a written code designating the patient to group D or S. Patients in Group D received oral esomeprazole (Nexium®, AstraZeneca AB, Södertälje, Sweden) 20 mg twice daily, and those in Group S received 20 mg once daily, respectively, for the following 36 weeks. The investigator who generated the random allocation sequence was different from those who assigned the participants to interventions and checked the symptoms and signs of recurrent bleeding during long-term follow-up. The latter were blinded to the study group allocation.

After the 52-week therapy, the patients in groups D and S used oral PPIs or did not at the discretion of their physicians according to their clinical needs. Thus, these patients were divided into a PPI-on-demand group and the PPI-discontinued group. The definition of PPI-discontinued was PPI use ≤3 days per week and ≤ 1 week per month. The others were defined as PPI-on-demand.

### Procedures

The patients with *Helicobacter pylori* (*H. pylori*) infection received standard first-line or second-line eradication and all were confirmed to have successful eradication by the ^13^C-urea breath test [18, 19]. During the periods of follow-up for primary and secondary outcomes, the patients in the three groups did not use gastric mucosal protective agents, including misoprostol and sucralfate. Bismuth was used for *H. pylori* eradication only.

### Outcomes

The primary and secondary outcomes were the recurrent peptic ulcer bleeding during the 1st year and the second year-and-thereafter since the first bleeding episode, respectively. All of the patients were monitored every day during hospitalization, every 2 weeks in the following 4 weeks, and every 12 weeks for the remaining weeks for a total of 1 year. They were monitored for pill counts, hemoglobin concentrations, and clinical symptoms and signs which were relevant to gastrointestinal bleeding, diarrhea, pneumonia, and bone fracture at each outpatient visit. All enrolled patients were included in the intention-to-treat (ITT) analysis, but patients who were lost to follow-up, discontinued intervention because of any reasons, had a protocol violation, or died were excluded from the per-protocol (PP) analysis for the primary endpoint. After the first year of therapy, the patients were continued to be monitored at outpatient departments every 12 weeks.

Recurrent bleeding was defined as follows, including 1) the presence of hematemesis, melena, hematochezia, or bloody aspirates through a nasogastric tube, plus 2) the presence of hemodynamic instability, including systolic blood pressure < 90 mmHg, heart rate > 120 beats per minute, or a drop in hemoglobin concentration by more than 2 g/dL, or sudden increase in transfusion requirements. To confirm recurrent peptic ulcer bleeding, the hemoglobin concentration and gastroscopy were performed to check the drop in hemoglobin concentration, any blood or coffee-ground-like materials in the stomach, or the recurrence of stigmata of recent hemorrhage. The gastroscopy also confirmed whether bleeding was from a peptic ulcer or other non-ulcer lesions, such as varices. Additionally, the definition of refractory or recurrence of the ulcer was the size ≥0.5 cm.

Medical events, including diarrhea and pneumonia, were monitored when patients took PPIs until 2 weeks after discontinuing PPIs. The definition of diarrhea was that the presence of loose or watery stools ≥ three times a day lasted for 1 day at least. The definition of pneumonia was the presence of one of the symptoms and signs of fever, purulent productive cough, and shortness of breath plus a typical infiltrative patch on chest X-ray. Additionally, any bone fracture, including a partial or complete break in the bone, was monitored until the last follow-up date at outpatient departments.

### Statistical analysis

The estimated recurrent bleeding rate during the first year in Group C was about 15% based on the previous studies [20, 21]. We proposed that the recurrent bleeding rate in the experiment groups could be reduced to near 2%, equal to that in *H. pylori* ulcers after eradication. The ratio of the patient number in each experiment group to the patient number in the control group was 2:5. With a two-side α value of 0.05 and power of 80% (β = 0.20), the number of patients required was 54 in each experiment group and 135 in the control group to detect a difference between the two groups. Assuming the rate of loss follow-up was 10%, sixty patients in each experimental group were enrolled. Data related to baseline characteristics and endpoints were evaluated using the one-way analysis of variance, Pearson’s χ2 test, Fisher’s exact test, or Kruskal-Wallis one-way analysis of variance by ranks. The log-rank test was used to compare the Kaplan-Meier curves among the study groups. The COX hazard regression analysis was used to evaluate the factors predicting the primary and secondary outcomes. When patients encountered death, no more chances of recurrent bleeding would be met. Thus, considering the patient’s death as a competing event, the proportional subdistribution hazards model for competing risk data according to the Fine and Gray method was used. All tests were two-tailed and *P* values of < 0.05 indicated significant differences. The statistical analysis was performed with SPSS software (SPSS Statistics 20.0, IBM Corp., Armonk, NY, USA) and the competing-risks regression was evaluated using SAS software (version 9.4, SAS Institute Inc., Cary, NC, USA).

## Results

### Demographic features of the patients

A total of 321 patients had peptic ulcer bleeding with major SRH and Rockall scores ≥ 6 were potentially eligible. Two hundred and one patients were excluded and the reasons for exclusion are described in detail in Fig. 1. Among the remaining 120 patients, they were randomized into either Group D (*n* = 60) or Group S (n = 60). The 135 patients from our previous cohort were matched into Group C (Fig. 1).

There were no significant differences in the baseline characteristics between the three study groups. However, the proportions of the Rockall scores and ASA physical status classification were significantly different between the three groups (*P* = 0.03 and 0.046) (Table 1).

**Table 1:** Baseline characteristics of the experimental groups and controls

Parameters Mean ± SD; n (%)	Group D (*n* = 60)	Group S (*n* = 60)	Group C (*n* = 135)	*P*^a^
Mean age (year)	71.0 ± 13.2	69.8 ± 12.9	70.5 ± 13.6	0.88
Female	23 (38.3)	22 (36.7)	50 (37.0)	0.98
Ulcer characteristics				
Gastric ulcer (n)	30 (50.0)	38 (63.3)	79 (58.5)	0.32
Mean ulcer size (cm)	1.21 ± 0.77	1.36 ± 0.87	1.30 ± 1.03	0.70
Forrest classification, Ia: Ib: IIa: IIb	0: 21 (35):30 (50): 9 (15)	3 (5): 13 (21.7): 36 (60): 8 (13.3)	3 (2.2): 28 (20.7): 81 (60): 23 (17)	0.37
Rockall scores 6: 7: 8: 9: 10	20 (33.3): 33 (55): 5 (8.3): 1 (1.7): 1 (1.7)	24 (40): 15 (25): 14 (23.3): 7 (11.7): 0 (0)	36 (26.7): 48 (35.6): 37 (27.4): 13 (9.6): 1 (0.7)	0.03
ASA physical status classification, class I: II: III: IV	0:10 (16.7):46 (76.7): 4 (6.7)	0:13 (21.7):43 (71.7):4 (6.7)	0:45 (33.3):83 (61.5):7 (5.2)	0.046
Cirrhosis	8 (13.3)	7 (11.7)	23 (17.0)	0.58
End-stage renal disease with maintenance dialysis	8 (13.3)	12 (20.0)	17 (12.6)	0.38
*H. pylori* infection^b^	12/50 (24.0)	18/55 (32.7)	45/117 (38.5)	0.19
Anti-coagulant use after enrollment	5 (8.3)	4 (6.7)	3 (2.2)	0.13
Mean hemoglobin at arrival (g/dL)	8.8 ± 2.6	8.3 ± 2.4	8.8 ± 2.6	0.33
Platelet counts < 50 × 10^9^/L at arrival	1 (1.7)	3 (5.0)	2 (1.5)	0.30
PT prolong ≥ 4 seconds at arrival	8 (13.3)	5 (8.3)	14 (10.4)	0.67
APTT prolong ≥ 1.5-fold at arrival	2 (3.3)	3 (5.0)	2 (1.5)	0.36
Serum albumin < 3.0 g/dL at arrival	17 (28.3)	15 (25.0)	42 (31.1)	0.68

Albumin: normal range 3.0–5.0 g/dL. Hemoglobin: normal range 13.5–17.0 g/dL. Platelet: normal range: 138–353 × 10^9^/L

*Abbreviations*: *APTT* activated partial thromboplastin time, *ASA* American Society of Anesthesiology, *H. pylori Helicobacter pylori*, *PT* prothrombin time, *SD* standard deviation

^a^The one-way analysis of variance, Pearson’s chi-square test, or Kruskal-Wallis one-way analysis of variance by ranks

^b^Some patients did not receive *H. pylori* infection survey because they were in a critical situation at the primary endoscopy

### Mortality and loss to follow-up in the three groups

As shown in Fig. 1, a total of 16 patients in Group D and Group S, respectively, and 6 patients in Group C were excluded from the per-protocol analysis of the primary outcome.

The causes of mortality included terminal hepatoma (*n* = 2) and terminal ovary cancer (*n* = 1) in Group D and multiple organ failure (*n* = 1) in Group S. In addition to 6 patients in Group C who died and were excluded from the per-protocol analysis, there were another 5 patients who had recurrent bleeding and died thereafter during the first year. The causes of mortality in Group C were stages IV-V chronic kidney disease with complications (*n* = 3), terminal cirrhosis (*n* = 1), septic shock in old stroke (*n* = 1), and disseminated cancers (*n* = 6). All-cause mortality rates were not significantly different between the three groups (5% [3/60] vs. 1.7% [1/60] vs. 8.1% [11/135], *P* = 0.20).

### Ulcer healing after the initial 16-week and medical events during the follow-up periods

There were 42 patients in Group D and 48 patients in Group S who received surveillance endoscopy after the initial 16-week PPIs (70.0% vs. 80.0%, *P* = 0.21). Among these patients, the rates of the non-healed ulcers (size ≥ 0.5 cm) were not significantly different between the two groups (9.5% [4/42] vs. 14.6% [7/48], *P* = 0.47).

During the follow-up periods, either the first year or the second year-and-thereafter, there were 1, 6, and 2 patients who had pneumonia, and 3, 2, and 12 had a bone fracture in Group D, S, and C, respectively. Additionally, 1 in Group S had pneumonia and bone fracture and 3 in Group C had diarrhea. The rates of overall medical events in the three groups were not significantly different (Group D vs. S vs. C, 6.7% [4/60] vs. 15.0% [9/60] vs. 12.6% [17/135], *P* = 0.33).

### The primary outcome, the recurrent peptic ulcer bleeding during the first year

During the first year, none in Group D, one in Group S, and 16 patients in Group C had recurrent peptic ulcer bleeding, respectively (Table 2). Eleven (64.7%) of them had recurrent bleeding ulcers in the same sites as the original bleeding. Thus, the rebleeding-free rates were significantly higher in Group D than in Group C (ITT analysis, *P* = 0.003; PP analysis, *P* = 0.01). The rebleeding-free rates were significantly higher in Group S than in Group C by ITT analysis (*P* = 0.02), but borderline higher by PP analysis (*P* = 0.08). The rebleeding-free rates were not significantly different between Group D and Group S (ITT and PP analyses, both *P* > 0.99).

**Table 2:** The cumulative between-group recurrent bleeding-free rates during the first year and the second year-and-thereafter

Rebleeding-free rates of peptic ulcer during the first year, N (%)	Group D (*n* = 60)	Group S (*n* = 60)	Group C (*n* = 135)	Group D *vs.* C		Group S *vs.* C		Group D *vs.* S	
				Relative risk (95% CI)	*P*^a^	Relative risk (95% CI)	*P*^a^	Relative risk (95% CI)	*P*^a^
*ITT analysis*	60/60 (100)	59/60 (98.3)	119/135 (88.1)	1.13 (1.07–1.21)	0.003	1.12 (1.04–1.20)	0.02	1.02 (0.98–1.05)	> 0.99
*PP analysis*	44/44 (100)	43/44 (97.7)	113/129 (87.6)	1.14 (1.07–1.22)	0.01	1.12 (1.03–1.21)	0.08	1.02 (0.98–1.07)	> 0.99
Rebleeding-free rates of peptic ulcer during the second year-and-thereafter, N (%)	PPI-on-demand subgroup (*n* = 32)	PPI-discontinued subgroup (*n* = 77)	Group C (*n* = 116)	PPI-on-demand *vs*. Group C		PPI-discontinued *vs*. Group C		PPI-on-demand *vs*. PPI-discontinued	
				Relative risk (95% CI)	*P*^a^	Relative risk (95% CI)	*P*^a^	Relative risk (95% CI)	*P*^a^
*ITT analysis*	30/32 (93.8)	66/77 (85.7)	92/116 (79.3)	1.18 (1.04–1.34)	0.06	1.08 (0.95–1.23)	0.26	1.09 (0.96–1.24)	0.34

*Abbreviations*: *ASA* American Society of Anesthesiology, *CI* confidence interval, *ITT* intention-to-treat, *PP* per protocol

^a^Pearson’s chi-square test or Fisher’s exact test was used with a 2-tailed analysis

**Fig. 2:**
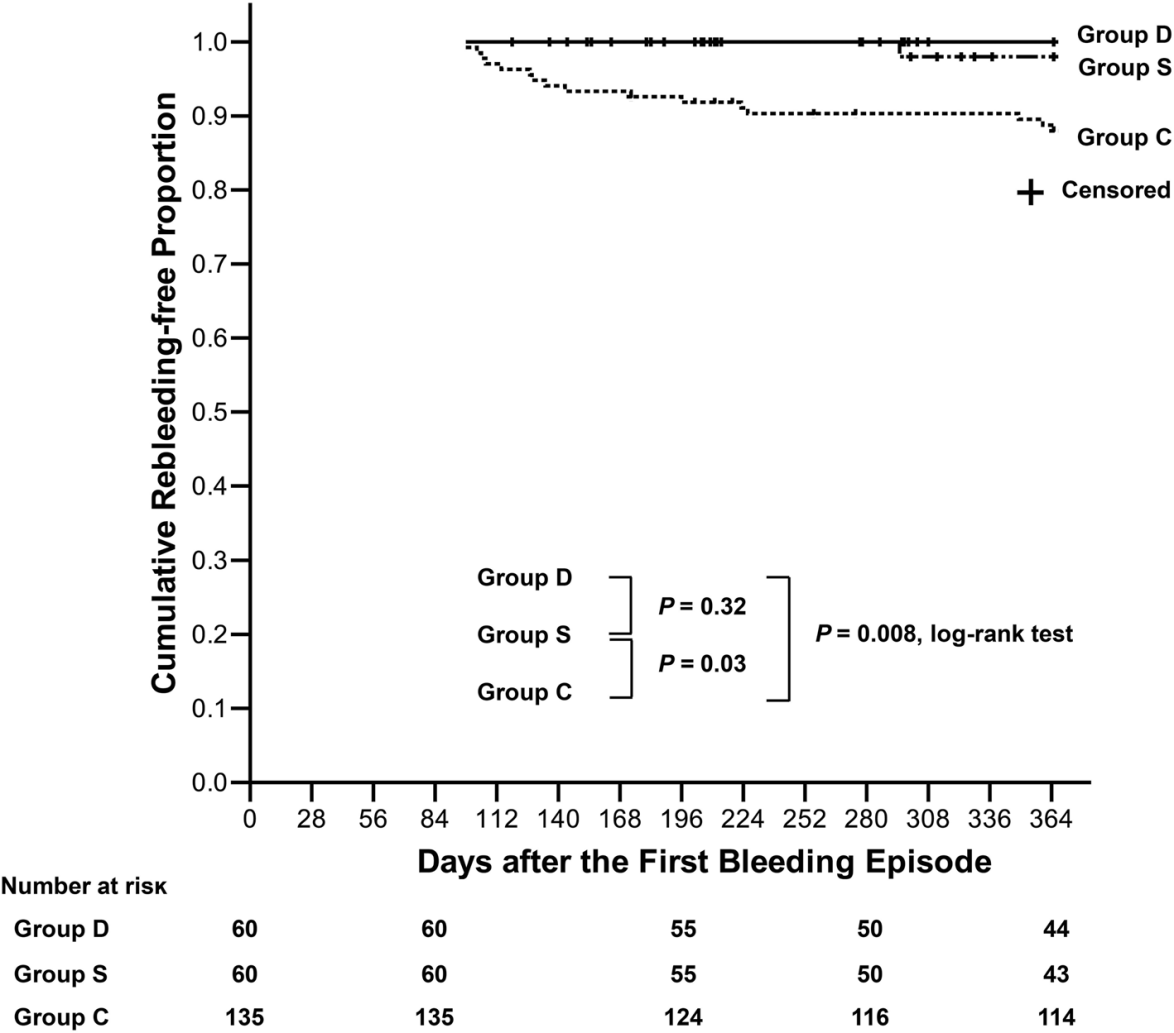
The cumulative rebleeding-free proportion during the first year. The Kaplan-Meier curves show that the cumulative proportions of patients who were bleeding-free during the first year were higher in Group D than in Group C and higher in Group S than in Group C (*P* = 0.008 and 0.03, log-rank test, respectively), but were not significantly different between Group D and Group S (*P* = 0.32, log-rank test)

In Fig. 2, the Kaplan-Meier curves showed the proportion of recurrent bleeding-free during the first year was higher in Group D than in Group C (*P* = 0.008, log-rank test) but not significantly different compared with Group S (*P* = 0.32). Moreover, the proportion of recurrent bleeding-free was higher in Group S compared with Group C (*P* = 0.03, log-rank test).

### The secondary outcome, the recurrent peptic ulcer bleeding during the second year-and-thereafter

During the second year-and-thereafter, eleven patients in Group D and S were excluded because lost to follow-up (*n* = 7) or having died (*n* = 4); thus, a total of 109 patients were still monitored. Among them, thirty-two patients received oral PPIs on-demand and 77 patients (43 from Group D and 34 from Group S) discontinued the experimental medication and did not take any other PPIs. In addition, 116 patients in Group C continued to be monitored during this period after excluding those lost to follow-up (*n* = 1), PPIs use (n = 7) and those having died during the first year (*n* = 11, Fig. 1).

The median (interquartile range [IQR], 25th–75th percentile) follow-up period from the first bleeding episode was 1650 (1090.8–1882.5) days in the PPI-on-demand group, 900 (582–1465.5) days in the PPI-discontinued group, and 1728.5 (658–2645.5) days in Group C, respectively (PPI-on-demand vs. PPI-discontinued, *P* < 0.001; PPI-on-demand vs. Group C, *P* = 0.35; and PPI-discontinued vs. Group C, *P* < 0.001, by Mann-Whitney U test).

**Fig. 3:**
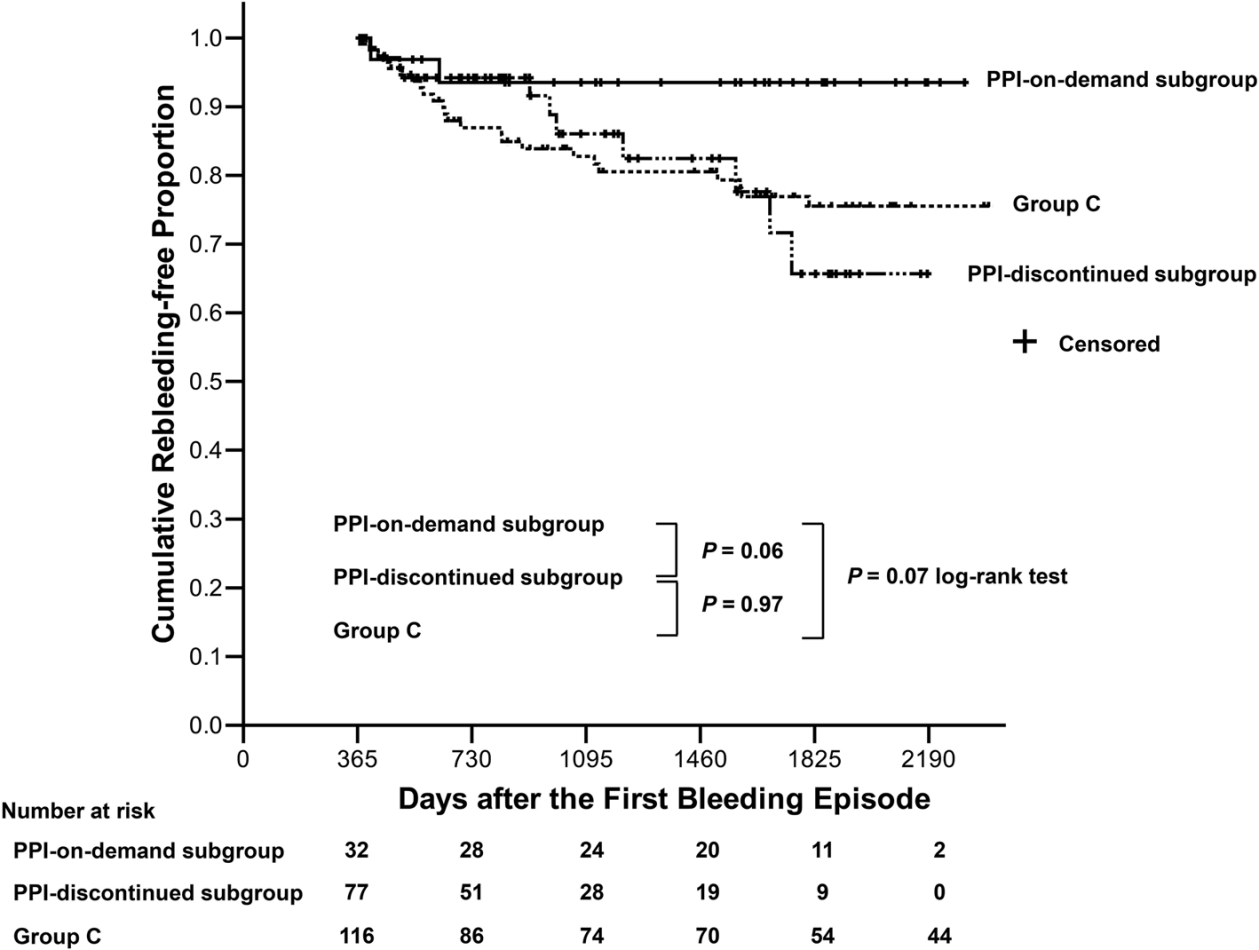
The cumulative rebleeding-free proportion during the second year-and-thereafter. The Kaplan-Meier curves show that the cumulative proportion of patients who were bleeding-free during the second year-and-thereafter was borderline higher in the PPI-on-demand subgroup as compared with those in the PPI-discontinued subgroup (*P* = 0.06, log-rank test) and in Group C (*P* = 0.07, log-rank test). However, the cumulative proportion of recurrent bleeding-free patients was not significantly different between the PPI-discontinued subgroup and Group C (*P* = 0.97, log-rank test). Abbreviations: PPI, proton pump inhibitor

Eventually, two patients (6.3%) among the patients with PPIs on-demand, 11 (14.3%) in the PPI-discontinued group, and 24 (20.7%) in Group C had recurrent peptic ulcer bleeding during the second year-and-thereafter (Table 2). The recurrent bleeding sites in 20 (54.1%) patients of them were the same as the original bleeding sites. The cumulative proportion of rebleeding-free patients was borderline higher in the PPI-on-demand subgroup than in the PPI-discontinued subgroup and in Group C (Fig. 3, *P* = 0.06 and 0.07, log-rank test, respectively), but there was not a significant difference between the PPI-discontinued subgroup and Group C (*P* = 0.97, log-rank test).

### The independent factors predicting the primary and secondary outcomes

Among the patients who were diagnosed with *H. pylori* infection at enrollment (Table 1), eleven (91.7%) in Group D, 17 (94.4%) in Group S, and 40 (88.9%) in Group C received successful *H. pylori* eradication (*P* = 0.78). Finally, a total of seven patients did not receive *H. pylori* eradication; moreover, none of them had recurrent bleeding either during the first year or during the second year-and-thereafter.

The COX hazard regression model was used to evaluate the independent factors predicting the primary outcome. The PPI use in groups D and S was combined to serve as the extended PPI use group. The univariable analysis showed the ASA classification, Rockall scores, and extended PPI use correlated to the primary outcome. Furthermore, the COX hazard regression analyses proved that the ASA classification and extended PPI use were two independent factors contributing to the primary outcome in the ITT and PP analyses (Table 3). After adjusting for the competing event, death, based on the cumulative incidence function, the proportional subdistribution hazards model showed that the ASA classification (*P* = 0.003) and extended PPI use (*P* = 0.03) remained as the independent factors.

**Table 3:** The independent factors correlated with the primary and secondary outcomes

Variables	Univariable analyses		Multivariable analyses	
	Hazard ratio (95% CI)	*P*^a^	Hazard ratio (95% CI)	*P*^a^
Primary outcome^b^, ITT				
Ages	1.00 (0.97–1.04)	0.81	–	–
Males	1.06 (0.42–2.96)	0.91	–	–
ASA classification	2.84 (1.15–6.94)	0.02	2.65 (1.04–6.65)	0.04
The Rockall score	1.72 (1.10–2.67)	0.02	1.31 (0.77–2.18)	0.31
The extended PPI use^c^	0.10 (0.01–0.42)	0.01	0.10 (0.01–0.41)	0.01
Primary outcome^b^, PP				
Ages	1.00 (0.97–1.04)	0.83	–	–
Males	1.05 (0.41–2.92)	0.93	–	–
ASA classification	3.13 (1.28–7.59)	0.01	2.59 (1.04–6.43)	0.04
The Rockall score	1.79 (1.14–2.82)	0.01	1.36 (0.80–2.27)	0.25
The extended PPI use^c^	0.13 (0.01–0.50)	0.02	0.13 (0.01–0.52)	0.02
Primary outcome^b^, CRR				
Ages	1.00 (0.96–1.05)	0.94	–	–
Males	1.85 (0.51–6.72)	0.35	–	–
ASA classification	2.22 (1.67–2.96)	<0.001	2.14 (1.29–3.55)	0.003
The Rockall score	1.59 (1.06–2.37)	0.03	1.26 (0.74–2.15)	0.40
The extended PPI use^c^	0.11 (0.01–0.85)	0.04	0.11 (0.01–0.84)	0.03
Secondary outcome^b^, ITT				
Ages	0.99 (0.96–1.01)	0.24	–	–
Males	1.35 (0.68–2.87)	0.42	–	–
ASA classification	1.10 (0.61–2.01)	0.75	–	–
The Rockall score	1.74 (1.25–2.42)	0.001	1.74 (1.25–2.44)	0.001
PPI-on-demand (*vs.* Group C)	0.35 (0.07–1.07)	0.12	0.38 (0.08–1.16)	0.15
PPI-discontinued (*vs.* Group C)	0.99 (0.47–1.97)	0.98	1.10 (0.52–2.21)	0.79
Secondary outcome^b^, CRR				
Ages	0.98 (0.96–1.01)	0.25	–	–
Males	1.19 (0.52–2.73)	0.67	–	–
ASA classification	1.18 (0.68–2.04)	0.56	–	–
The Rockall score	1.52 (1.07–2.15)	0.02	1.53 (1.07–2.18)	0.02
PPI-on-demand (*vs.* Group C)	0.47 (0.10–2.08)	0.32	0.52 (0.12–2.34)	0.39
PPI-discontinued (*vs.* Group C)	1.24 (0.53–2.15)	0.61	1.36 (0.57–3.24)	0.48

*Abbreviations*: *ASA* American Society of Anesthesiologists, *CI* confidence interval, *CRR* competing-risks regression, *ITT* intention-to-treat, *PP* per-protocol, *PPI* proton pump inhibitor

^a^The COX hazard regression model or the competing risks regression analysis

^b^The primary and secondary outcomes were recurrent peptic ulcer bleeding during the 1^st^ year and during the second year-and-thereafter after the first bleeding episode, respectively

^c^The extended PPI use included PPI use in groups D and S

For the secondary outcome, the multivariable COX hazard regression and the proportional subdistribution hazards model showed that the Rockall score was the only independent factor (*P* = 0.001 and 0.02, respectively, Table 3).

## Discussion

This study demonstrated that an extended course of oral esomeprazole 20 mg for 36 weeks in either a twice- or once-daily manner after an initial 16-week PPI therapy could protect patients with Rockall scores ≥ 6 from delayed recurrent peptic ulcer bleeding. However, such protective effects tended to diminish after PPIs were discontinued thereafter.

Although a 4- to 8-week treatment of PPIs can heal more than 90% of peptic ulcers [22], there were still patients who were at risk of recurrent peptic ulcer bleeding over the subsequent years [21, 23, 24]. *H. pylori* infection is one of the major causes of recurrent peptic ulcers, and *H. pylori* eradication should be performed if *H. pylori* infection is confirmed [18, 19]. In this study, nearly 90% of *H. pylori*-infected patients received *H. pylori* eradication. Only seven patients didn’t receive eradication therapy; however, none of them encountered recurrent ulcer bleeding during the follow-up periods. Nevertheless, over the past decades, *H. pylori*-negative peptic ulcer has become an emerging issue because of the high recurrent bleeding risk [21, 23, 25]. Patients with *H. pylori*-negative ulcers were elderly and had underlying comorbidities [21, 23, 26]; therefore, such patients were prone to have high Rockall scores. Rockall scores ≥ 6 are a prognostic score for peptic ulcer bleeding in a 3.5-year follow-up [8]. In this study, the healing rates of ulcers in such patients after an initial 16-week PPI therapy were as high as 87.8% (79/90). However, there are few studies exploring a prolonged course of PPIs to reduce the risk of long-term recurrent bleeding among such high-risk patients. This study showed that as compared to the controls, the risk of recurrent bleeding during the first year was reduced by an extended 36-week course of oral esomeprazole 20 mg twice or once daily (Fig. 2). Once-daily dosing of oral PPIs may not suppress acid secretion over a complete 24-hour period, but twice-daily dosing of PPIs extends the mean residence time [10, 13, 14, 27]. However, this study did not show a significant difference in terms of risk reduction between twice- and once-daily esomeprazole. The sample size may be small to detect difference. Nevertheless, the COX hazard regression model and the competing-risks regression analysis confirmed that the ASA classification and extended PPI use were two independent factors during the first year (Table 3).

During the second year-and-thereafter, the COX hazard regression model or the competing-risks regression analyses showed that only the Rockall score was correlated with recurrent bleeding, and not on-demand PPIs or PPI discontinuation (Table 3). These findings reinforced that these patients were still at high risk of recurrent peptic ulcer bleeding if PPI therapy was shifted to on-demand or discontinued. Two previous studies showed that regular acid-suppressive therapy not prescribed at the discretion of the physician reduced the risk of recurrent peptic ulcer bleeding among high-risk patients [28, 29]. All of these showed that the risk of recurrent bleeding could be prevented by oral PPIs; however, the shortest possible duration of PPI usage remained uncertain. Studies with a longer course or even lifelong use of oral PPIs to evaluate the benefit and risks are needed.

There is concern about the benefits and risks of long-term PPI use [15, 30, 31]. The medical events possibly correlated with PPI use include diarrhea, pneumonia, and bone fracture, and the rates were similar between the three groups in the present study. An extended course of oral esomeprazole did not increase such medical events. However, due to worldwide population aging and increases in the proportion of patients with a high risk of recurrent peptic ulcer bleeding [23, 26], a strategy to reduce such risk is needed. Therefore, the findings of the present study provide evidence of the benefits and risks associated with an extended course of oral PPIs in such patients and are generalizable to clinical practice worldwide.

The present study had limitations. First, we did not design a parallel-control group to receive a placebo because of ethics concerns. Nevertheless, we used the data from a historical control group. The controls had similar baseline characteristics except for the distribution of Rockall scores and the ASA classification. After adjusting for these two possible confounders, extended PPI use was an independent factor by which to predict the primary outcome. Thus, the bias due to a non-parallel-group design for the controls was reduced. Second, the dropout rate in the experimental groups was higher than expected because the PPI use was interrupted by multiple medical events. Nevertheless, the significant between-group differences in the primary outcomes decreased the concern of a type II error. Third, the sample size was limited to evaluate the secondary outcome; thus, further study is needed. Fourth, genotyping tests for CYP2C19 alleles were not conducted in this study. Nevertheless, all groups shared a similar fair condition. Moreover, no change in dosing of esomeprazole was recommended for various CYP2C19 metabolizers because evidence of different treatment outcome was insufficient [32].

## Conclusions

In conclusion, patients with Rockall scores ≥6 using an extended course of oral esomeprazole 20 mg in an either twice- or once-daily manner up to 1 year after ulcer bleeding exhibited a lower cumulative risk of recurrent ulcer bleeding as compared to those using an 8- to 16-week course of oral PPIs. However, the protective effect of reducing recurrent peptic ulcer bleeding after shifting to on-demand or discontinuing the use of PPIs needed to be further validated.

## Supplementary Information

**Additional file 1: Supplementary Table 1.** The parameters of the Rockall score.

**Additional file 2: Supplementary Table 2.** American Society of Anesthesiologists Physical Status classification system.

## Data Availability

The datasets used during the current study are available from the corresponding author upon reasonable request.

## Abbreviations

APTT: Activated partial thromboplastin time
ASA: American Society of Anesthesiologists
BPM: Beats per minute
CI: Confidence interval
CRR: Competing-risks regression
*H. pylori*: *Helicobacter pylori*
IQR: Interquartile range
ITT: Intention-to-treat
PP: Per protocol
PPI: Proton pump inhibitor
PT: Prothrombin time
SBP: Systolic blood pressure
SD: Standard deviation
SRH: Stigmata of recent hemorrhage.
