# Supplementary material for: An extended 36-week oral esomeprazole improved long-term recurrent peptic ulcer bleeding in patients at high risk of rebleeding
Source: BMC Gastroenterol. 2022 Oct 21;22:439. doi: 10.1186/s12876-022-02534-0 (PMC9585769; doi:10.1186/s12876-022-02534-0)
Supplement: Supplementary file 1 — Additional file 1: Supplementary Table 1. The parameters of the Rockall score. [file 12876_2022_2534_MOESM1_ESM.docx]

| Risk parameters | Definition | Score |
| --- | --- | --- |
| Age (years) | < 60 | 0 |
|  | 60–79 | 1 |
|  | ≥ 80 | 2 |
| Shock | No shock (pulse rate < 100 bpm and SBP ≥ 100 mmHg) | 0 |
|  | Tachycardia (pulse rate ≥ 100 bpm and SBP ≥ 100 mmHg) | 1 |
|  | Hypotension (SBP < 100 mmHg) | 2 |
| Comorbidity | No comorbidity | 0 |
|  | Comorbidities, including ischemic heart disease, cardiac failure, or any others | 2 |
|  | Renal failure, liver failure, or metastatic cancer | 3 |
| Endoscopic diagnosis | Mallory-Weiss tear or no lesion and without bleeding | 0 |
|  | All other diagnosis | 1 |
|  | Malignancy of upper gastrointestinal tract | 2 |
| Endoscopic evidence of bleeding | No stigmata of recent hemorrhage or only dark spot in ulcer base | 0 |
|  | Blood in upper gastrointestinal tract, adherent clot, visible or spurting vessel | 2 |

The table was modified according to the 6^th^ and 7^th^ references. Abbreviations: BPM, beats per minute; SBP, systolic blood pressure.
