# Supplementary material for: An extended 36-week oral esomeprazole improved long-term recurrent peptic ulcer bleeding in patients at high risk of rebleeding
Source: BMC Gastroenterol. 2022 Oct 21;22:439. doi: 10.1186/s12876-022-02534-0 (PMC9585769; doi:10.1186/s12876-022-02534-0)
Supplement: Supplementary file 2 — Additional file 2: Supplementary Table 2. American Society of Anesthesiologists Physical Status classification system. [file 12876_2022_2534_MOESM2_ESM.docx]

| ASA Physical Status classification | Definition | Examples |
| --- | --- | --- |
| Class 1 | A normal healthy patient | Nonsmoking, no or minimal drinking, and without organic, physiologic, biochemical, or psychiatric disturbance |
| Class 2 | A patient with mild systemic disturbance of distress but without functional limitations | Non- or only slightly limiting organic heart disease, mild and well-controlled hypertension/diabetes, anemia, obesity (30 < BMI < 40), or chronic bronchitis |
| Class 3 | A patient with moderate to severe systemic disease or disturbance with substantive functional limitations | Active hepatitis, severely limiting organic heart disease, coronary artery disease/stents, history (> 3 months) myocardial infarction/cerebral vascular accident, moderate reduction of ejection fraction, severe, poor-controlled, or with vascular complications of hypertension/diabetes, moderate to severe pulmonary insufficiency, or end-stage renal disease with maintenance dialysis, morbid obesity (BMI ≥ 40) |
| Class 4 | A patient with severe, life-threatening systemic disorders | Advanced hepatic insufficiency, organic heart disease with signs of cardiac insufficiency, recent (< 3 months) myocardial infarction/cerebral vascular accident, persistent angina, severe reduction of ejection fraction, active myocarditis, advanced endocrine insufficiency, acute respiratory distress syndrome, end-stage renal disease without maintenance dialysis, shock, sepsis, or disseminated intravascular coagulation |
| Class 5 | A moribund patient who has little chance of survival without the operation | Ruptured aortic aneurysm with shock, massive trauma, intracranial bleeding with mass effect, ischemic bowel with multiple organ failure |
| Class 6 | A patient who is declared brain-dead for organ donation | A cadaver donor |

The table was modified according to the 17^th^ reference. Abbreviations: ASA, American Society of Anesthesiologists; BMI, body mass index.
